# Prevalence, Sex Differences, and Predictors of Internet Gaming Disorder Among Impoverished Rural Adolescents: Cross-Sectional and Prospective Cohort Study

**DOI:** 10.2196/83522

**Published:** 2025-11-17

**Authors:** Chenhan Wang, Yifan Li, Shuhong Lin, Qiuping Huang, Yongyan Shi, Guangxian Yang, Erjia Huang, Xicheng Deng, Jinwen Luo

**Affiliations:** 1 The Affiliated Children's Hospital of Xiangya School of Medicine (Hunan Children’s Hospital), Central South University, No. 86 Ziyuan Road, Yuhua District, Hunan Province, Changsha, 410007, China, 86 073185600966; 2 Department of Psychiatry, The Second Xiangya Hospital of Central South University, Changsha, China; 3 Department of Psychology, School of Humanities and Management, Hunan University of Chinese Medicine, Changsha, China; 4 Clinical Nursing Teaching and Research Section, The Second Xiangya Hospital, Central South University, Changsha, China

**Keywords:** internet gaming disorder, rural adolescents, prevalence, sex differences, longitudinal study, adolescent mental health

## Abstract

**Background:**

Internet gaming disorder (IGD) is prevalent globally and linked to significant negative outcomes. Impoverished rural adolescents face unique risks due to limited supervision and unequal digital resources, with limited longitudinal research conducted in this population. Existing studies show sex differences in IGD prevalence, but their manifestations and mechanisms in rural populations remain unclear.

**Objective:**

This is the first large-sample cross-sectional and prospective cohort study targeting impoverished adolescents in rural areas. It aimed to determine the prevalence of IGD among impoverished rural adolescents, identify sex-specific risk and protective factors, and evaluate the longitudinal predictors of IGD.

**Methods:**

In this study, self-administered questionnaires were used to collect demographic characteristics, affective states, impulsivity, gaming time, and scores for IGD. First, the prevalence of IGD at baseline and follow-up, along with sex differences, was calculated. Correlation analysis was conducted to explore variables associated with IGD. Subsequently, multivariate logistic regression analysis was conducted to identify baseline and follow-up predictors of IGD.

**Results:**

The cross-sectional analysis at baseline included 13,931 valid responses (IGD prevalence: n=725, 5.2%; males: 489/7304, 6.7%; females: 236/6627, 3.6%). A 1-year longitudinal follow-up showed IGD prevalence of 5% (692/13,931; males: 511/7304, 7.0%; females: 181/6627, 2.7%; after multiple imputations). Common baseline factors were gaming time (females: odds ratio [OR] 1.11, 95% CI 1.08-1.14, *P*<.001; males: OR 1.11, 95% CI 1.09-1.13, *P*<.001), self-esteem scores (females: OR 0.95, 95% CI 0.92-0.98, *P*=.002; males: OR 0.95, 95% CI 0.92-0.97, *P*<.001), depression scores (females: OR 1.14, 95% CI 1.11-1.16, *P*<.001; males: OR 1.11, 95% CI 1.09-1.13, *P*<.001), and impulsive behavior (females: OR 1.16, 95% CI 1.10-1.22, *P*<.001; males: OR 1.10, 95% CI 1.06-1.14, *P*<.001). Companionship (OR 0.71, 95% CI 0.52-0.97; *P*=.03) was a protective factor for females, while age (OR 1.08, 95% CI 1.02-1.15; *P*=.02) and poor self-regulation (OR 1.07, 95% CI 1.03-1.11; *P*=.001) posed extra risk for males at baseline. Longitudinal predictors were baseline gaming time (females: OR 1.06, 95% CI 1.03-1.09, *P*<.001; males: OR 1.02, 95% CI 1.00-1.05, *P*=.03) and impulsive behavior (females: OR 1.38, 95% CI 1.30-1.46, *P*<.001; males: OR 1.27, 95% CI 1.22-1.31, *P*<.001). Baseline companionship (OR 0.32, 95% CI 0.23-0.43; *P*<.001) was a protective factor for females, while baseline poor self-regulation (OR 1.32, 95% CI 1.27-1.37; *P*<.001) was a predictive factor for males after 1 year.

**Conclusions:**

IGD prevalence was lower in rural than in urban populations and higher in males than in females. Impulsivity, gaming time, and guardian companionship showed sex differences: females relied more on companionship, whereas males were more vulnerable to poor self-regulation. Interventions should address these differences, strengthening family support and psychological adjustment. This study provides novel insights into sex-specific pathways of IGD in rural settings and offers empirical evidence for developing targeted prevention strategies, highlighting its practical significance for public health.

## Introduction

In 2019, the World Health Organization officially included gaming disorder in the *International Classification of Diseases, 11th Revision*, identifying its core features as a shift in gaming priority, loss of behavioral control, and impairment in social functioning. According to the latest meta-analyses, the prevalence of internet gaming disorder (IGD) among adolescents in East Asia is approximately 6%, with a rate of approximately 6.4% reported in China [1,2].

To date, most research on IGD has focused on urban populations. However, children and adolescents in economically underdeveloped rural areas may face unique challenges such as limited parental supervision resources, lack of guidance on digital device use, and unequal distribution of digital resources between urban and rural areas. These environmental differences may contribute to distinct risk profiles for IGD among rural youth compared to their urban counterparts [3]. Given that IGD has been linked to negative outcomes, including poorer sleep quality, impaired affective regulation, decreased attention in learning, and increased risk of suicidal ideation [4-7], the underlying mechanisms of IGD may be closely associated with reward system dysfunction and impaired cognitive control abilities [8,9]. Thus, systematically assessing the epidemiology and risk mechanisms of IGD in rural areas holds great public health importance. Unfortunately, existing studies targeting rural populations are mostly cross-sectional, which only allow for identification of baseline associations but fall short of revealing temporal dynamics or causal relationships. For example, it remains unclear whether mental health issues lead to IGD, whether IGD triggers subsequent problems, or whether both exhibit a bidirectional relationship among rural adolescents. This limitation greatly restricts the applicability and scalability of current intervention strategies in rural contexts.

Considerable evidence indicates sex differences in the prevalence of IGD, with male individuals typically exhibiting higher rates than female individuals [10,11], but the underlying mechanisms remain insufficiently understood. Historically, higher prevalence in male individuals has been partly attributed to the predominance of male-targeted game designs that emphasize stimulation and control; however, the market is increasingly offering games designed to cater to female players, and the number of male and female gamers is now broadly comparable [12]. This indicates that differences in prevalence cannot be fully explained by game types and motivation design alone. Current research is shifting focus toward potential individual susceptibility factors. For instance, male individuals may exhibit greater vulnerability in impulsivity and reward sensitivity, whereas being female may be more closely associated with difficulties in emotional regulation [13,14]. Crucial questions that remain unanswered include whether the sex disparity observed in previous studies persists in rural samples, whether female students with subthreshold baseline gaming problems are at elevated risk of progression over time, and whether factors such as family support and affective symptoms exert differential effects by sex. These questions warrant targeted longitudinal investigation in rural school-aged populations to clarify sex-specific pathways and inform tailored preventive interventions.

This study aimed to determine the prevalence of IGD among impoverished rural primary and middle school students in China, identify sex-specific risk and protective factors, and evaluate longitudinal predictors of IGD over a 1-year follow-up period. We hypothesized that (1) the prevalence of IGD in this rural school-aged cohort would differ from findings in broader or urban-representative samples; (2) individuals with longer baseline gaming time, more severe depressive state, and higher impulsivity would be more likely to develop IGD subsequently; (3) higher baseline self-esteem and greater guardian or parental involvement would be associated with lower risk of IGD; and (4) predictive factor patterns would differ by sex. The findings of this longitudinal study are expected to provide empirical evidence to inform precise prevention and intervention strategies for IGD in rural schools.

## Methods

### Study Design and Data Collection Process

This study adopted a cross-sectional and prospective longitudinal cohort design and was conducted and reported in accordance with the Strengthening the Reporting of Observational Studies in Epidemiology statement [15]. The completed Strengthening the Reporting of Observational Studies in Epidemiology checklist is provided as Checklist 1. From February 2023 to July 2024, the baseline survey was conducted in primary and middle schools across 13 impoverished rural counties in Hunan province, as well as in the rural free clinic program of Hunan Children’s Hospital. As data collection was bound to the free clinic schedule, no a priori sample size calculation was conducted; instead, all eligible students were enrolled, yielding 18,967 valid questionnaires. Baseline data were obtained via on-site paper questionnaires covering demographics, self-esteem, affective status, impulsivity, gaming time, and IGD.

Follow-up began in January 2024 among students who completed the baseline survey and whose schools arranged a second free clinic. Using the same questionnaire method, follow-up continued until June 2025, with 5254 responses collected. Loss to follow-up mainly reflected schools that had not yet organized a second clinic, resulting in a nonrandom attrition pattern. Identity checks were used to ensure accurate matching of baseline and follow-up data.

### Participants

Participants were recruited through their respective schools to respond to on-site surveys by distributing questionnaires collectively in class groups during the free medical consultation event.

The inclusion criteria for this study were age between 6 and 18 years; long-term residence in impoverished rural areas of Hunan province; regular attendance to rural primary or secondary schools; and voluntary participation of both the student and their primary guardian, with signed informed consent.

Exclusion criteria included the presence of cognitive or language impairments preventing comprehension or completion of the questionnaire, temporary attendance to rural schools for less than 6 months, and refusal to participate or withdrawal during questionnaire completion for personal reasons.

### Measurements

A self-developed questionnaire was used to collect demographic information, including age, sex, years of education, only child status, and daily time spent playing internet games (hours per day). The questionnaire also assessed the quality of the relationship between the participants and their primary guardians (rated as very poor, poor, good, or very good), as well as whether the primary guardian was able to provide companionship when needed (hereinafter referred to as *companionship*).

The Chinese version of the Rosenberg Self-Esteem Scale was used to assess overall self-esteem. This scale consists of 10 items and uses a 4-point Likert scale for scoring, with higher scores indicating higher levels of overall self-esteem. The scale’s Cronbach α was 0.88 [16]. The Patient Health Questionnaire–9 (PHQ-9) was used to assess depressive mood. This scale includes 9 items and uses a 4-point Likert scale for scoring, with higher total scores reflecting more severe depressive mood. The scale’s Cronbach α was 0.90 [17,18]. The Generalized Anxiety Disorder–7 (GAD-7) scale was used to assess anxious mood. This scale comprises 7 items and uses a 4-point Likert scale for scoring, with higher total scores reflecting more severe anxious mood. The scale’s Cronbach α was 0.92 [19]. The Brief Barratt Impulsiveness Scale (BBIS) was used to assess impulsivity. This scale includes 8 items and uses a 4-point Likert scale for scoring. The scale covers 2 dimensions: poor self-regulation and impulsive behavior. Higher scores indicate greater impulsivity. The Cronbach α was 0.85 for the total scale and 0.88 and 0.80 for the 2 subscales [20]. The Internet Gaming Disorder Scale–Short Form (IGDS9-SF) was used to assess IGD. The scale contains 9 items and uses a 5-point Likert scale for scoring, with higher scores indicating more severe IGD symptoms. The scale’s Cronbach α was 0.91 [21]. In this study, participants scoring >32 on the IGDS9-SF were classified as having IGD.

### Quality Control

Before the assessments at each school, 5 professional psychiatrists provided standardized training to the teachers. The training covered the study objectives, interpretation of questionnaire items, informed consent procedures, on-site guidance protocols, and handling of abnormal situations, enabling teachers to assist psychiatrists in guiding students during questionnaire completion.

To ensure data quality, questionnaires were excluded if they met any of the following criteria: (1) careless responding—defined as 3 or more scales having all valid items scored identically or exhibition of systematic repetitive response patterns (eg, 1-2-3-4, 4-3-2-1, or 1-2-1) on any core scale; (2) failed validity check items—the questionnaires included lie detection items requiring the response “most of the time,” and deviations from this response were deemed invalid; (3) implausible key information (age outside the range of 6 to 18 years; gaming time outside 0 to 24 hours; missing data for age, sex, years of education, or gaming time; or inconsistency between age and years of education); and (4) more than 10% of items left unanswered.

A total of 6452 invalid questionnaires were excluded. Data entry was conducted using EpiData (version 3.1; EpiData Association). To ensure accuracy, 30% of entered questionnaires were randomly rechecked, maintaining an error rate below 1%.

### Handling of Missing Data

As the follow-up survey of this study only targeted students whose schools subsequently arranged a second free medical consultation, there were cases of missing data. To assess the mechanism of missing data, this study adopted the Little missing completely at random (MCAR) test. If the *P* value of this test is less than .05, the null hypothesis that the data are MCAR is rejected. On this basis, to further use all baseline information and reduce potential bias, this study used the *mice* package in the R language (R Foundation for Statistical Computing) to perform multiple imputation for the missing data. All statistical analyses related to the status of IGD during the follow-up period—including IGD prevalence rate, persistence rate, and the longitudinal predictors of IGD—were conducted based on the multiple imputation datasets, whereas the cross-sectional analyses involving only baseline data were completed using the original observed data.

### Statistical Analysis

Statistical analyses were conducted using SPSS (version 26.0; IBM Corp ). The prevalence of IGD was calculated for the total sample and by sex at baseline and follow-up. The Kolmogorov-Smirnov test was used to assess the normality of the continuous variables. Categorical variables (eg, only child status and relationship with guardian) were analyzed using chi-square tests. Normally distributed continuous variables (eg, age and PHQ-9 scores) were compared using independent-sample *t* tests, whereas nonnormally distributed variables were analyzed using the Mann-Whitney *U* test. Furthermore, the false discovery rate (FDR) multiple-testing (Benjamini-Hochberg) correction method was used to control the false positive rate of differences in variable comparisons between different-sex individuals at baseline, and a postcorrection *P* value of less than .05 was considered statistically significant. The Spearman rank correlation was used to examine associations between IGD indicators and other variables. Multivariate logistic regression was performed to identify factors associated with baseline IGD and predict follow-up IGD. First, baseline IGD status was set as the dependent variable, with variables showing significance in univariate analyses included as independent variables to identify associated factors. Next, follow-up IGD status was set as the dependent variable, with baseline IGD and its independent associated factors included as predictors to explore risk factors. The statistical significance level for correlation and regression was set at *P*<.05 for 2-tailed tests.

### Ethical Considerations

This study was conducted in accordance with the Declaration of Helsinki as set forth by the World Medical Association. Ethics approval was obtained from the institutional review board of the Hunan Children’s Hospital affiliated with Xiangya School of Medicine, Central South University (KS2025-169). This study was part of a free medical outreach program in impoverished rural areas, so no financial compensation was provided to participants. Before data collection, teachers explained the questionnaire content to participants and their primary guardians. The first section of the questionnaire included informed consent information, and participants or their guardians were free to decline participation or withdraw at any time without penalty. All participants provided written informed consent. The informed consent for the primary data collection complied with the institutional review board’s guidelines and permitted secondary data analysis without the need for additional consent.

While the original data contained personal identifiers, all participants’ names were hidden during the analysis phase to ensure confidentiality. Data were securely stored in password-protected systems accessible only to authorized researchers. No identification of individual participants or users in any images of the manuscript or supplementary material is possible.

## Results

### Prevalence of IGD and Sex Differences

**Figure 1.**
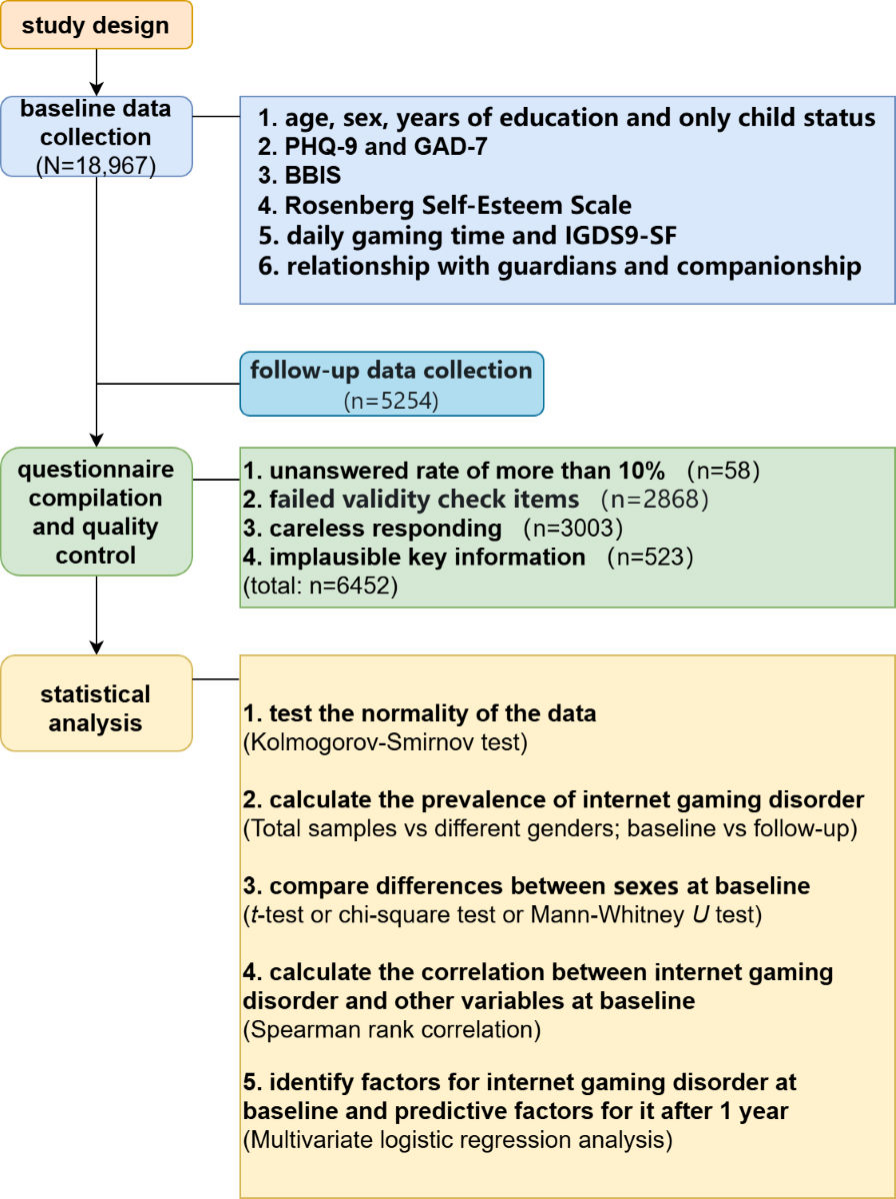
Flowchart. BBIS: Brief Barratt Impulsiveness Scale; GAD-7: Generalized Anxiety Disorder–7; IGDS9-SF: Internet Gaming Disorder Scale–Short Form; PHQ-9: Patient Health Questionnaire–9.

A total of 18,967 baseline data entries were collected in this study. After quality control, 13,931 valid questionnaires were obtained for the baseline cross-sectional study; please refer to Figure 1 for the detailed screening process. These data revealed a prevalence of IGD of 5.2% (725/13,931; 95% CI 4.8%-5.6%). The prevalence among male individuals (489/7304, 6.7%; 95% CI 6.1%-7.3%) was significantly higher than that among female individuals (236/6627, 3.6%; 95% CI 3.1%-4%; *χ*^2^_1_=69.2; risk difference [RD] 0.31, 95% CI 0.02-0.04; *P*<.001).

For the 1-year follow-up assessment, 3838 valid questionnaires were obtained (loss to follow-up rate: 10,093/13,931, 72.4%; 95% CI 71.7%-73.2%). The result of the Little MCAR test indicated that the data were not MCAR (*χ*^2^_9_=1358.2, *P*<.001). The longitudinal data after multiple imputation showed that the overall prevalence of IGD was 5% (692/13,931; 95% CI 4.6%-5.3%), and the prevalence among male individuals remained significantly higher than that among female individuals (511/7304, 7%; 95% CI 6.4%-7.6% vs 181/6627, 2.7%; 95% CI 2.4%-3.2%, respectively; *χ*^2^_1_=133.9; RD 0.04, 95% CI 0.04-0.05; *P*<.001).

Among participants with IGD at baseline, the persistence rate at follow-up of IGD was 9.1% (66/725; 95% CI 7.2%-11.45%). Specifically, the persistence rate was 7.2% (17/236; 95% CI 4.5%-11.2%) in the female subgroup and 10% (49/489; 95% CI 7.7%-13.1%) in the male subgroup. There was no statistically significant difference between the 2 subgroups (*χ*^2^_1_=1.5; RD 0.03, 95% CI –0.02 to 0.07; *P*=.22).

As a sensitivity analysis, we also conducted the same analysis on the full-case data, yielding results that were largely consistent with the multiple imputation analysis (see Table S1 in Multimedia Appendix 1 for details).

### Sex Differences in Baseline Demographic and Clinical Variables

An analysis of baseline cross-sectional data revealed significant sex differences in several demographic and clinical variables. When grouped by sex, there was a higher proportion of male individuals who were only children than female individuals (*χ*^2^_1_=71.8, post-FDR *P*<.001; RD 0.06, 95% CI 0.04-0.07) and a greater percentage who reported “very good” relationships with their primary guardian (*Z=*–4.69; median difference [MD] 0.03, 95% CI 0.02-0.05; post-FDR *P*<.001) and longer daily gaming time (*Z=*–2.83; MD –0.23, 95% CI −0.35 to −0.10; post-FDR *P*=.008). Male individuals also scored higher on the IGDS9-SF (*Z=*–18.79; MD –2.20, 95% CI –2.45 to −1.95; post-FDR *P*<.001) and self-esteem (*t*_13,929_*=*–9.89; MD –0.81, 95% CI −0.97 to −0.65; post-FDR *P*<.001). Conversely, male individuals had significantly lower scores on the GAD-7 (*Z=*–13.57; MD 0.83, 95% CI 0.68-0.98; post-FDR *P*<.001), the PHQ-9 (*Z=*–9.27; MD 0.71, 95% CI 0.54-0.89; post-FDR *P*<.001), self-regulation (*t*_13,929_=3.81; MD 0.18, 95% CI 0.09-0.27; post-FDR *P*<.001), and the total BBIS impulsivity scale (*t*_13,929_=2.51; MD 0.18, 95% CI 0.04-0.32; post-FDR *P*=.01). For specific baseline variable differences between sexes, please refer to Table 1.

**Table 1.** Sex differences in baseline demographic characteristics, family relationship, anxiety, depression, self-esteem, impulsivity, gaming time, and internet gaming disorder scores. The Mann-Whitney *U* test after *z* transformation requires no calculation of df.

Variables	Female individuals (n=6627)	Male individuals (n=7304)	Statistics; Z score, t test, or chi-square (*df*)	Risk difference or median difference (95% CI)	Pre-FDR^a^ *P* value	Post-FDR *P* value
Age (y), mean (SD)	12.11 (1.57)	12.12 (1.57)	−0.26 (13,929)^b^	−0.01 (−0.06 to 0.04)	.80	.85
Education (y), mean (SD)	6.33 (1.58)	6.28 (1.58)	1.86 (13,929)^b^	0.05 (−0.003 to 0.10)	.06	.08
Only child, n (%)			71.75 (1)^c^	0.06 (0.04 to 0.07)	<.001	<.001
No	5525 (83.4)	5677 (77.7)				
Yes	1101 (16.6)	1627 (22.3)				
Relationship with primary guardian, n (%)			−4.69 (3)^d^	0.03 (0.02 to 0.05)	<.001	<.001
Very poor	25 (0.4)	43 (0.6)				
Poor	92 (1.4)	93 (1.3)				
Good	1709 (25.8)	1615 (22.1)				
Very good	4801 (72.4)	5553 (76.0)				
Companionship, n (%)			0.49 (1)^c^	0.004 (−0.01 to 0.02)	.48	.54
No	1137 (17.2)	1286 (17.6)				
Yes	5490 (82.8)	6018 (82.4)				
Gaming time (h per d), mean (SD)	2.60 (3.55)	2.83 (3.77)	−2.83 (13,929)^d^	−0.23 (−0.35 to −0.10)	.005	.008
Self-esteem score (range 10-40), mean (SD)	28.15 (4.91)	28.96 (4.75)	−9.89 (13,929)^b^	−0.81 (−0.97 to −0.65)	<.001	<.001
GAD-7^e^ score (range 0-21), mean (SD)	4.86 (4.48)	4.04 (4.39)	−13.57 (13,929)^d^	0.83 (0.68 to 0.98)	<.001	<.001
PHQ-9^f^ score (range 0-27), mean (SD)	5.37 (5.43)	4.66 (5.16)	−9.27 (13,929)^d^	0.71 (0.54 to 0.89)	<.001	<.001
IGDS9-SF^g^ score (range 9-45), mean (SD)	14.51 (6.91)	16.70 (7.97)	−18.79 (13,929)^d^	−2.20 (−2.45 to −1.95)	<.001	<.001
Poor self-regulation score (range 4-16), mean (SD)	9.17 (2.65)	9.00 (2.84)	3.81 (13,929)^b^	0.18 (0.09 to 0.27)	<.001	<.001
Impulsive behavior score (range 4-16), mean (SD)	8.20 (2.51)	8.20 (2.66)	0.03 (13,929)^b^	0.001 (−0.08 to 0.09)	.98	.98
BBIS^h^ score (range 8-32), mean (SD)	17.37 (4.13)	17.19 (4.26)	2.51 (13,929)^b^	0.18 (0.04 to 0.32)	.01	.01

a FDR: false discovery rate.

b *t* test.

c Chi-square test.

d Z score.

e GAD-7: Generalized Anxiety Disorder–7.

f PHQ-9: Patient Health Questionnaire–9.

g IGDS9-SF: Internet Gaming Disorder Scale–Short Form.

h BBIS: Brief Barratt Impulsiveness Scale.

### Correlation Analysis of IGD Indicators

**Figure 2.**
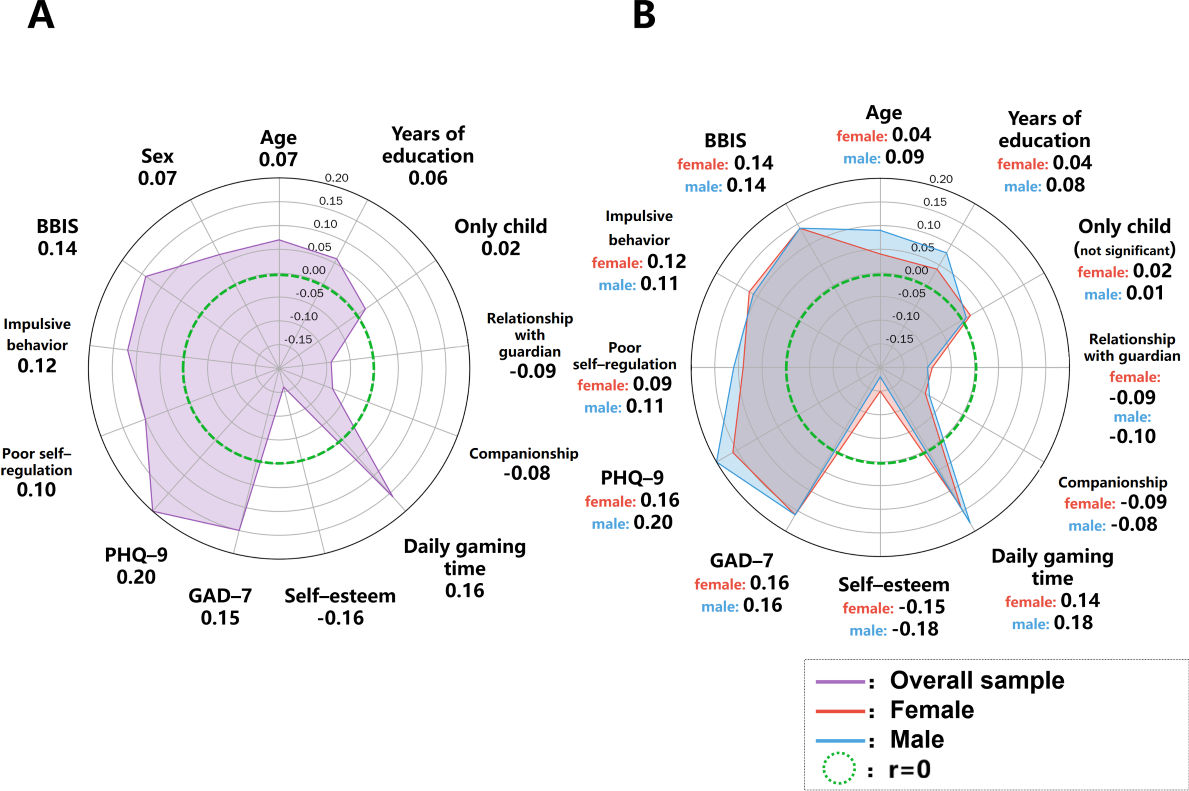
Variables associated with internet gaming disorder in (A) the baseline overall sample and (B) male and female individuals. BBIS: Brief Barratt Impulsiveness Scale; GAD-7: Generalized Anxiety Disorder–7; PHQ-9: Patient Health Questionnaire–9.

On the basis of the baseline cross-sectional data, IGD indicators were significantly correlated with multiple factors (Figure 2), including age (*r*=0.07, 95% CI 0.05-0.08; *P*<.001), years of education (*r*=0.06, 95% CI 0.04-0.08; *P*<.001), relationship with primary guardian (*r=*–0.09, 95% CI −0.11 to −0.07; *P*<.001), companionship (*r*=–0.08, 95% CI −0.10 to −0.06; *P*<.001), only child status (*r*=0.02, 95% CI 0.004-0.04; *P*=.02), gaming time (*r*=0.16, 95% CI 0.15-0.18; *P*<.001), poor self-regulation (*r*=0.10, 95% CI 0.08-0.12; *P*<.001), impulsive behavior (*r*=0.12, 95% CI 0.10-0.13; *P*<.001), BBIS score (*r*=0.14, 95% CI 0.12-0.15; *P*<.001), self-esteem (*r=*–0.16, 95% CI −0.17 to −0.14; *P*<.001), GAD-7 score (*r*=0.15, 95% CI 0.14-0.17; *P*<.001), and PHQ-9 score (*r*=0.20, 95% CI 0.18-0.21; *P*<.001).

Subgroup analyses by sex revealed that, for female individuals, there were significant correlations with age (*r*=0.04, 95% CI 0.02-0.06; *P*=.002), years of education (*r*=0.04, 95% CI 0.01-0.06; *P*=.005), relationship with primary guardian (*r=*–0.09, 95% CI −0.11 to −0.06; *P*<.001), companionship (*r*=–0.09, 95% CI −0.12 to −0.05; *P*<.001), gaming time (*r*=0.14, 95% CI 0.11-0.16; *P*<.001), self-esteem (*r*=–0.15, 95% CI −0.17 to −0.13; *P*<.001), GAD-7 score (*r*=0.16, 95% CI 0.14-0.18; *P*<.001), PHQ-9 score (*r*=0.16, 95% CI 0.14-0.17; *P*<.001), poor self-regulation (*r*=0.09, 95% CI 0.07-0.12; *P*<.001), impulsive behavior (*r*=0.12, 95% CI 0.10-0.15; *P*<.001), and BBIS score (*r*=0.14, 95% CI 0.11-0.16; *P*<.001). For male individuals, significant correlations were found with age (*r*=0.09, 95% CI 0.07-0.11; *P*<.001), years of education (*r*=0.08, 95% CI 0.06-0.10; *P*<.001), relationship with primary guardian (*r=*–0.10, 95% CI −0.12 to −0.07; *P*<.001), companionship (*r*=–0.08, 95% CI −0.11 to −0.06; *P*<.001), gaming time (*r*=0.18, 95% CI 0.16-0.21; *P*<.001), self-esteem (*r=*–0.18, 95% CI −0.20 to −0.15; *P*<.001), GAD-7 score (*r*=0.16, 95% CI 0.14-0.19; *P*<.001), PHQ-9 score (*r*=0.20, 95% CI 0.18-0.23; *P*<.001), poor self-regulation (*r*=0.11, 95% CI 0.09-0.13; *P*<.001), impulsive behavior (*r*=0.11, 95% CI 0.09-0.14; *P*<.001), and BBIS score (*r*=0.14, 95% CI 0.12-0.17; *P*<.001). Additionally, being male was positively correlated with IGD in the overall sample (*r*=0.07; *P*<.001).

### Identification of Factors Associated With IGD

#### Overview

**Figure 3.**
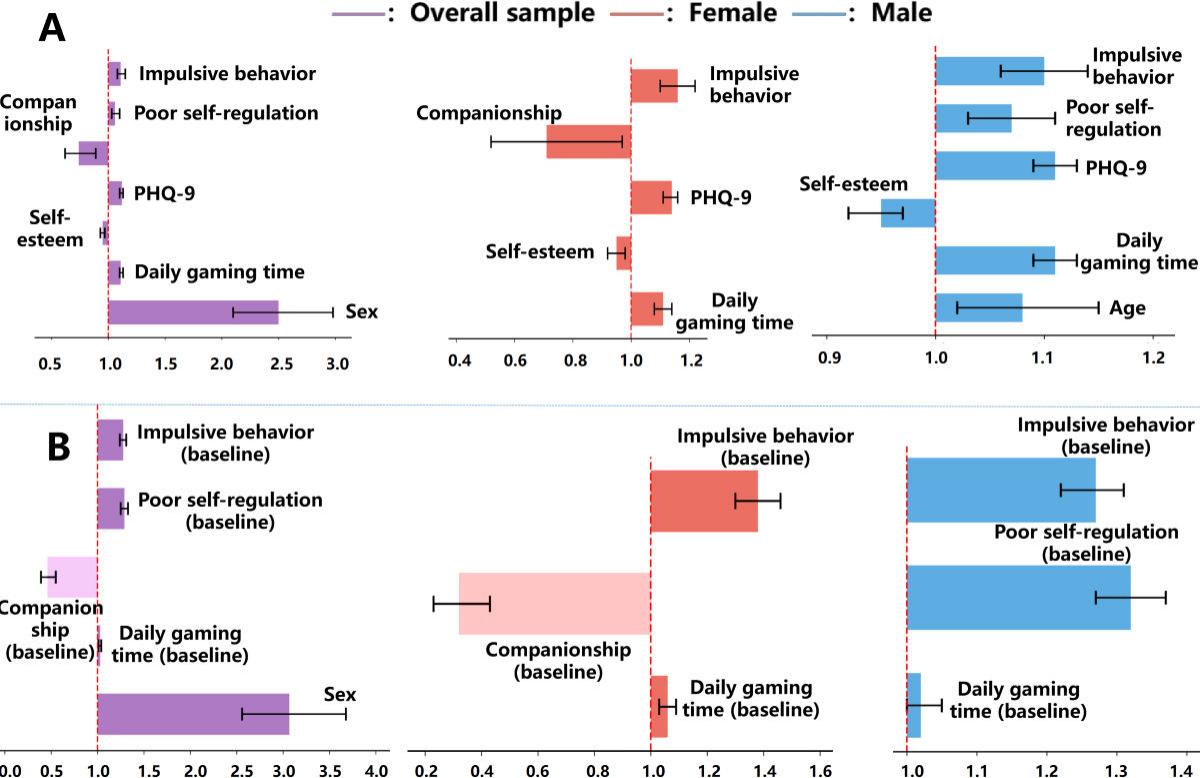
Odds ratios for (A) identifying baseline factors of internet gaming disorder and (B) predicting follow-up internet gaming disorder. PHQ-9: Patient Health Questionnaire–9.

Multivariate logistic regression models using backward stepwise selection (Wald test) were used, first, to identify factors cross-sectionally associated with IGD at baseline and, second, to longitudinally predict IGD case status at follow-up, as shown in Figure 3.

#### Baseline IGD Identification Factors (Cross-Sectional Analysis)

In the overall sample, male sex (odds ratio [OR] 2.50, 95% CI 2.10-2.98; *P<*.001), gaming time (OR 1.11, 95% CI 1.10-1.13; *P<*.001), PHQ-9 score (OR 1.12, 95% CI 1.10-1.13; *P<*.001), impulsive behavior (OR 1.11, 95% CI 1.08-1.15; *P<*.001), and self-regulation (OR 1.06, 95% CI 1.03-1.10; *P<*.001) were identified as risk factors. Protective factors included self-esteem score (OR 0.95, 95% CI 0.93-0.97; *P<*.001) and companionship (OR 0.74, 95% CI 0.62-0.89; *P=*.001).

In sex subgroup analyses, common cross-sectional identification factors for IGD included gaming time (female individuals: OR 1.11, 95% CI 1.08-1.14, *P<*.001; male individuals: OR 1.11, 95% CI 1.09-1.13, *P<*.001), self-esteem score (female individuals: OR 0.95, 95% CI 0.92-0.98, *P=*.002; male individuals: OR 0.95, 95% CI 0.92-0.97, *P<*.001), PHQ-9 score (female individuals: OR 1.14, 95% CI 1.11-1.16, *P<*.001; male individuals: OR 1.11, 95% CI 1.09-1.13, *P<*.001), and impulsive behavior (female individuals: OR 1.16, 95% CI 1.10-1.22, *P<*.001; male individuals: OR 1.10, 95% CI 1.06-1.14, *P<*.001). Differences included that companionship was a protective factor for female individuals (OR 0.71, 95% CI 0.52-0.97; *P=*.03), whereas age (OR 1.08, 95% CI 1.02-1.15; *P=*.02) and poor self-regulation (OR 1.07, 95% CI 1.03-1.11; *P=*.001) were additional risk factors for male individuals.

#### Predictors of IGD at the 1-Year Follow-Up (Longitudinal Analysis)

This study then examined baseline variables as longitudinal predictors of developing IGD at follow-up. In the overall sample, male sex (OR 3.07, 95% CI 2.56-3.68; *P<*.001), baseline gaming time (OR 1.03, 95% CI 1.01-1.04; *P=*.005), baseline poor self-regulation (OR 1.29, 95% CI 1.25-1.33; *P<*.001), and baseline impulsive behavior (OR 1.28, 95% CI 1.24-1.31; *P<*.001) were risk factors. Protective factors included companionship at baseline (OR 0.46, 95% CI 0.39-0.55; *P<*.001).

In sex subgroup analyses, common longitudinal predictors included baseline gaming time (female individuals: OR 1.06, 95% CI 1.03-1.09, *P<*.001; male individuals: OR 1.02, 95% CI 1.00-1.05, *P=*.03) and baseline impulsive behavior (female individuals: OR 1.38, 95% CI 1.30-1.46, *P<*.001; male individuals: OR 1.27, 95% CI 1.22-1.31, *P<*.001). Differences included that companionship at baseline was a protective predictor for female individuals (OR 0.32, 95% CI 0.23-0.43; *P<*.001), whereas poor self-regulation at baseline (OR 1.32, 95% CI 1.27-1.37; *P<*.001) was a predictor for male individuals.

## Discussion

### Principal Findings

To our knowledge, this study is the first large-scale longitudinal investigation in China focusing on the prevalence, influencing factors, and sex differences regarding IGD among rural adolescents, and it provides new evidence for intervention strategies targeting IGD among adolescents in impoverished rural areas. This pioneering work fills a critical gap in rural mental health research and has practical implications for designing tailored interventions. Key findings indicate that, in the overall sample, the baseline prevalence of IGD was 5.2% (725/13,931), with a 1-year follow-up prevalence of 5% (692/13,931). Both analyses revealed significantly higher prevalence rates among male individuals than among female individuals. There were common cross-sex risk factors for IGD at both the baseline and follow-up stages: longer gaming time and greater impulsivity behaviors. Sex-specific differences were primarily observed in protective factors and long-term predictive effects—guardian companionship exhibited a significant protective effect for female individuals, whereas poor self-regulation at baseline was a unique long-term predictor for male individuals. Excluding self-esteem and depressive states, these findings align with the 4 hypotheses of this study.

The baseline (725/13,931, 5.2%) and 1-year follow-up (692/13,931, 5%) prevalence rates of IGD among rural students in this study were notably lower than the 10% reported in urban Chinese samples [22]. This discrepancy may be attributed to limited internet infrastructure, fewer gaming devices, and economic constraints restricting frequent gaming in rural areas. Regarding sex differences, male individuals consistently exhibited significantly higher prevalence rates across all stages, aligning with most previous research [23]. This pattern may reflect male individuals’ greater preference for competitive, task-oriented games and gaming motivations centered on achievement and self-affirmation [24].

Individual psychological characteristics demonstrated stable effects in identifying or predicting IGD. Depressive symptoms were significant risk factors across sex subgroups at baseline [25], supporting the “emotion escape model” [26], which suggests that individuals with negative emotions such as depression or anxiety may turn to online activities such as gaming as a way to temporarily escape these feelings. This maladaptive coping strategy can contribute to the development of IGD over time. Impulsivity and poor self-regulation were significantly associated with both cross-sectional and longitudinal outcomes, consistent with the interaction of person-affect-cognition-execution model [27]. The interaction of the person-affect-cognition-execution model proposes that addictive behaviors, including IGD, arise from the interaction of personal characteristics (eg, impulsivity), affective responses, cognitive factors, and executive functioning deficits. These factors interact to reduce an individual’s ability to resist the immediate rewards offered by gaming, thereby increasing the likelihood of developing a disorder.

Self-esteem served as a cross-sex protective factor at baseline, particularly pronounced in female individuals. Individuals with higher self-esteem may exhibit greater psychological resilience when facing peer pressure or identity challenges, reducing reliance on gaming for self-worth—corroborating the buffering effect of self-esteem in adolescent internet addiction [28]. Thus, psychological interventions targeting affective regulation, self-regulation, and self-evaluation may offer effective pathways for preventing and treating IGD. Notably, after multiple imputation of the entire baseline sample, baseline self-esteem no longer significantly predicted follow-up IGD, suggesting selective bias in the previous complete case analysis. The association patterns between low self-esteem and IGD differed between the follow-up sample and the sample lost to follow-up, and multiple imputation corrected this bias by estimating the IGD status of those lost to follow-up. Evidently, self-esteem’s long-term predictive power for IGD is less direct and stable than in cross-sectional studies, potentially mediated or masked by factors such as impulsivity and family companionship.

Gaming time significantly predicted IGD across sex subgroups, consistent with previous studies [29,30]. Excessive gaming not only increases exposure to virtual environments but also diminishes real-life social and emotional interactions, reinforcing immediate reward mechanisms and deepening immersion [27], thereby intensifying dependence. Over time, the weakening of real-world functions may lead individuals to increasingly turn to gaming as an escape from stress, which in turn drives longer gaming sessions, creating a closed loop of addictive behavior. Therefore, targeted interventions to control gaming time should not only reduce opportunities for triggering addictive behavior at its source but also create conditions for individuals to rebuild real-world social connections and restore a sense of purpose, thereby lowering the risk of developing and progressing into IGD.

Guardian companionship emerged as a significant protective factor at both baseline and follow-up, especially for female adolescents. Stable emotional support and supervision reduce adolescents’ reliance on virtual worlds, consistent with family functioning research indicating that positive parent-child interactions, emotional support, and behavioral monitoring lower the risk of internet addiction [31,32]. For impoverished rural students, the importance of caregiver companionship is even more pronounced: rural left-behind children commonly experience emotional support deficits [33], and unmet emotional needs may drive compensatory gaming behaviors where immediate feedback and a sense of belonging reinforce use. This finding aligns with attachment theory, which posits that secure attachment reduces dependence on alternative emotional sources. For female adolescents, lack of companionship significantly elevates risk, underscoring the buffering role of emotional support [34].

This study highlights significant sex differences in predictors of IGD. For female adolescents, caregiver companionship was the most salient protective factor, indicating that familial emotional support and presence effectively mitigate the risk of gaming dependence when vulnerability arises. Female individuals with access to emotional support, particularly during adversity, may better regulate negative emotions and reduce the likelihood of gaming addiction [34]. Conversely, poor self-regulation was a significant risk predictor for male adolescents. Due to higher extraversion and lower self-regulation, male individuals are more prone to engaging in high-stimulation, immediate-feedback gaming activities, increasing IGD risk [35]. This pattern aligns with male behavioral tendencies to regulate emotions through external stimulation [36]. The distinct pathways observed between male and female individuals suggest that future research and intervention strategies should fully consider sex differences, avoiding one-size-fits-all approaches.

### Limitations

There were several limitations to this study. First, all data relied on self-report, which may introduce social desirability bias (underreporting gaming time to avoid disapproval) and recall bias (inaccurate memory of past gaming behaviors), affecting data reliability. Second, the limited number of assessment scales restricted the measurement of key factors such as family parenting styles and school interventions, leaving potential associations with IGD unexamined. Third, a single 1-year follow-up failed to capture long-term dynamic changes in IGD and the sustained effects of influencing factors. Fourth, nonrandom sampling (relying on medical programs and government-recommended schools) may limit the generalizability of the results to a broader population of impoverished rural adolescents. Fifth, a second free medical consultation was not arranged in some regions, resulting in the loss of follow-up data. This may limit, to a certain extent, the generalizability of this study’s conclusions to adolescents in regions without follow-up. Future research could address these limitations by integrating objective data (eg, mobile phone use logs) to verify self-reports, expanding the scales to include parenting- and school-related variables, increasing follow-up frequency and duration, and adopting random sampling to improve representativeness.

### Conclusions

In summary, this pioneering large-scale prospective longitudinal study provides robust evidence on the prevalence and sex-specific predictors of IGD among impoverished rural adolescents in China. The findings reveal that IGD prevalence in rural areas is lower than that in urban regions, with male adolescents exhibiting significantly higher rates than their female counterparts. Impulsivity, gaming time, and caregiver companionship emerged as key influencing factors, demonstrating notable sex differences in both protective and risk profiles. Specifically, female individuals benefited more from the protective role of caregiver companionship, whereas male individuals were more vulnerable to deficits in self-regulation. These findings hold significant practical implications, underscoring the necessity of developing tailored public health strategies that address sex-specific needs, strengthen family support systems, and enhance psychological resilience to effectively prevent and mitigate IGD in these vulnerable communities. Future research should integrate multisource data and conduct long-term follow-ups to deepen understanding of IGD development and refine intervention approaches.

## Supplementary material

10.2196/83522 Multimedia Appendix 1 Identification factors of internet gaming disorder under baseline conditions, predictive factors of internet gaming disorder under follow-up conditions.

10.2196/83522 Checklist 1 STROBE checklist.

## Abbreviations

BBIS: Brief Barratt Impulsiveness Scale
FDR: false discovery rate
GAD-7: Generalized Anxiety Disorder–7
IGD: internet gaming disorder
IGDS9-SF: Internet Gaming Disorder Scale–Short Form
MCAR: missing completely at random
MD: median difference
OR: odds ratio
PHQ-9: Patient Health Questionnaire–9
RD: risk difference
STROBE: Strengthening the Reporting of Observational Studies in Epidemiology
