# Supplementary material for: Prevalence, Sex Differences, and Predictors of Internet Gaming Disorder Among Impoverished Rural Adolescents: Cross-Sectional and Prospective Cohort Study
Source: JMIR Serious Games. 2025 Nov 17;13:e83522. doi: 10.2196/83522 (PMC12622857; doi:10.2196/83522)
Supplement: Multimedia Appendix 1 [file games-v13-e83522-s001.docx]

**1. Table S1 Comparison of the prevalence rates (overall/male/female) and persistence rates of IGD in one-year follow-up data before and after multiple imputation**

| Item |  | Before multiple imputation | After multiple imputation |
| --- | --- | --- | --- |
| Sample size | overrall sample | 3838 | 13931 |
|  | female sample | 1946 | 6627 |
|  | male sample | 1892 | 7304 |
| Prevalence of IGD | overrall sample | 5.6% 215/3838 | 5.0% 692/13931 |
|  | female sample | 3.4% 67/1946 | 2.7% 181/6627 |
|  | male sample | 7.8%148/1892 | 7.0% 511/7304 |
| Difference in prevalence rates between male and female samples | *χ^2^* | 34.80 | 133.88 |
|  | *P* | <.001 | <.001 |
| Persistence of IGD | overrall sample | 7.5% 17/226 | 9.1% 66/725 |
|  | female sample | 9.0% 8/89 | 7.2% 17/236 |
|  | male sample | 5.8% 9/154 | 10.0% 49/489 |
| Difference in persistence rates between male and female samples | *χ^2^* | 0.86 | 1.53 |
|  | *P* | .36 | .22 |

NOTE: IGD = Internet gaming disorder

**2. Identification Factors of Internet Gaming Disorder Under Baseline Conditions (without multiple imputation analysis)**

Taking the presence or absence of Internet Gaming Disorder (IGD) as the dependent variable, and regarding factors significantly correlated with IGD as independent variables, multiple logistic regression models (backward method, Wald criterion) were constructed respectively in the total sample and the male and female subgroups to explore the identification factors of IGD.

**Table S2 Identification Factors of Internet Gaming Disorder (IGD) in the Total Sample Under Baseline Conditions without multiple imputation analysis**

| Variables | β | Wald | *P value* | OR | 95%CI | VIF |
| --- | --- | --- | --- | --- | --- | --- |
| Sex (male) | 0.92 | 106.88 | <.001 | 2.50 | 2.10-2.98 | 1.01 |
| gaming time | 0.11 | 219.47 | <.001 | 1.11 | 1.10-1.13 | 1.06 |
| Self-esteem scores | -0.05 | 29.49 | <.001 | 0.95 | 0.93-0.97 | 1.46 |
| PHQ-9 scores | 0.11 | 238.69 | <.001 | 1.12 | 1.10-1.13 | 1.44 |
| Companionship | -0.30 | 10.19 | .001 | 0.74 | 0.62-0.89 | 1.05 |
| Poor self-regulation | 0.06 | 14.85 | <.001 | 1.06 | 1.03-1.10 | 1.16 |
| Impulsive behavior | 0.11 | 45.38 | <.001 | 1.11 | 1.08-1.15 | 1.12 |

**Table S3 Identification Factors of Internet Gaming Disorder (IGD) in the Female Adolescent Sample Under Baseline Conditions without multiple imputation analysis**

| Variables | β | Wald | *P value* | OR | 95%CI | VIF |
| --- | --- | --- | --- | --- | --- | --- |
| gaming time | 0.11 | 71.14 | <.001 | 1.11 | 1.08-1.14 | 1.06 |
| Self-esteem scores | -0.05 | 9.78 | .002 | 0.95 | 0.92-0.98 | 1.40 |
| PHQ-9 scores | 0.13 | 108.76 | <.001 | 1.14 | 1.11-1.16 | 1.46 |
| Companionship | -0.34 | 4.74 | .03 | 0.71 | 0.52-0.97 | 1.06 |
| Impulsive behavior | 0.15 | 27.08 | <.001 | 1.16 | 1.10-1.22 | 1.11 |

**Table S4 Identification Factors of Internet Gaming Disorder (IGD) in the Male Adolescent Sample Under Baseline Conditions without multiple imputation analysis**

| Variables | β | Wald | *P value* | OR | 95%CI | VIF |
| --- | --- | --- | --- | --- | --- | --- |
| Age | 0.08 | 5.80 | .02 | 1.08 | 1.02-1.15 | 1.10 |
| gaming time | 0.11 | 125.48 | <.001 | 1.11 | 1.09-1.13 | 1.13 |
| Self-esteem scores | -0.06 | 21.20 | <.001 | 0.95 | 0.92-0.97 | 1.45 |
| PHQ-9 scores | 0.10 | 124.68 | <.001 | 1.11 | 1.09-1.13 | 1.41 |
| Companionship | -0.22 | 3.48 | .06 | 0.80 | 0.64-1.01 | 1.05 |
| Poor self-regulation | 0.06 | 11.39 | .001 | 1.07 | 1.03-1.11 | 1.15 |
| Impulsive behavior | 0.09 | 21.89 | <.001 | 1.10 | 1.06-1.14 | 1.10 |

**3. Predictive Factors of Internet Gaming Disorder (IGD) Under Follow-Up Conditions (without multiple imputation analysis)**

With the presence or absence of IGD at the follow-up time point as the dependent variable, and taking the pre-test IGD status and the identification factors of IGD at baseline as independent variables, multiple logistic regression models (backward method, Wald criterion) were constructed respectively in the total sample and the male and female subgroups to explore the longitudinal predictive factors of IGD.

**Table S5 Predictive Factors of Internet Gaming Disorder (IGD) in the Total Sample Under Follow-Up Conditions without multiple imputation analysis**

| Variables | β | Wald | *P value* | OR | 95%CI | VIF |
| --- | --- | --- | --- | --- | --- | --- |
| Sex (male) | 1.12 | 146.91 | <.001 | 3.07 | 2.56-3.68 | 1.00 |
| Gaming time at baseline | 00.03 | 8.05 | .005 | 1.03 | 1.01-1.04 | 1.02 |
| Companionship at baseline | -0.77 | 76.87 | <.001 | 0.46 | 0.39-0.55 | 1.03 |
| Poor self-regulation at baseline | 0.26 | 262.32 | <.001 | 1.29 | 1.25-1.33 | 1.08 |
| Impulsive behavior at baseline | 0.24 | 242.99 | <.001 | 1.28 | 1.24-1.31 | 1.08 |

**Table S6 Predictive Factors of Internet Gaming Disorder (IGD) in the Female Adolescent Sample Under Follow-Up Conditions without multiple imputation analysis**

| Variables | β | Wald | *P value* | OR | 95%CI | VIF |
| --- | --- | --- | --- | --- | --- | --- |
| Gaming time at baseline | 0.06 | 13.02 | <.001 | 1.06 | 1.03-1.09 | 1.02 |
| Companionship at baseline | -1.15 | 52.65 | <.001 | 0.32 | 0.23-0.43 | 1.03 |
| Impulsive behavior at baseline | 0.32 | 124.64 | <.001 | 1.38 | 1.30-1.46 | 1.04 |

**Table S7 Predictive Factors of Internet Gaming Disorder (IGD) in the Male Adolescent Sample Under Follow-Up Conditions without multiple imputation analysis**

| Variables | β | Wald | *P value* | OR | 95%CI | VIF |
| --- | --- | --- | --- | --- | --- | --- |
| IGD at baseline | -0.29 | 2.66 | .10 | 0.75 | 0.53-1.06 | 1.07 |
| Gaming time at baseline | 0.02 | 4.54 | .03 | 1.02 | 1.00-1.05 | 1.07 |
| Poor self-regulation at baseline | 0.28 | 231.01 | <.001 | 1.32 | 1.27-1.37 | 1.06 |
| Impulsive behavior at baseline | 0.24 | 169.29 | <.001 | 1.27 | 1.22-1.31 | 1.06 |
